# Engineered T cells from induced pluripotent stem cells: from research towards clinical implementation

**DOI:** 10.3389/fimmu.2023.1325209

**Published:** 2024-01-12

**Authors:** Ratchapong Netsrithong, Laura Garcia-Perez, Maria Themeli

**Affiliations:** ^1^ Department of Hematology, Amsterdam University Medical Center (UMC), Vrije Universiteit Amsterdam, Amsterdam, Netherlands; ^2^ Cancer Biology and Immunology, Cancer Center Amsterdam, Amsterdam, Netherlands

**Keywords:** iPSC, CAR T cell, cGMP, quality control, scalability, hematopoietic progenitor, T cells

## Abstract

Induced pluripotent stem cell (iPSC)-derived T (iT) cells represent a groundbreaking frontier in adoptive cell therapies with engineered T cells, poised to overcome pivotal limitations associated with conventional manufacturing methods. iPSCs offer an off-the-shelf source of therapeutic T cells with the potential for infinite expansion and straightforward genetic manipulation to ensure hypo-immunogenicity and introduce specific therapeutic functions, such as antigen specificity through a chimeric antigen receptor (CAR). Importantly, genetic engineering of iPSC offers the benefit of generating fully modified clonal lines that are amenable to rigorous safety assessments. Critical to harnessing the potential of iT cells is the development of a robust and clinically compatible production process. Current protocols for genetic engineering as well as differentiation protocols designed to mirror human hematopoiesis and T cell development, vary in efficiency and often contain non-compliant components, thereby rendering them unsuitable for clinical implementation. This comprehensive review centers on the remarkable progress made over the last decade in generating functional engineered T cells from iPSCs. Emphasis is placed on alignment with good manufacturing practice (GMP) standards, scalability, safety measures and quality controls, which constitute the fundamental prerequisites for clinical application. In conclusion, the focus on iPSC as a source promises standardized, scalable, clinically relevant, and potentially safer production of engineered T cells. This groundbreaking approach holds the potential to extend hope to a broader spectrum of patients and diseases, leading in a new era in adoptive T cell therapy.

## 1 Introduction

Adoptive immunotherapy with engineered T cells is a significant therapeutic tool in the field of cancer, infectious diseases, autoimmune diseases and transplantation. More specifically, therapy with chimeric antigen receptor (CAR)-engineered T cells (CAR T) has demonstrated great potential in treating various hematological malignancies and is now commercially available for relapsed or refractory B-cell acute lymphoblastic leukemia (ALL), lymphomas and Multiple Myeloma (1). In addition, the use of CAR T cells or CAR-engineered regulatory T cells (CAR Treg) has shown pre-clinical and clinical utility for diseases beyond cancer such as autoimmune diseases (SLE, RA, MS etc) (2–4), graft-versus-host disease (5, 6), transplant rejection (7, 8). These results dictate for further advances allowing expanding the applicability of adoptive T cell therapy for more patients. Nevertheless, current manufacturing approaches for engineered T cells limit the feasibility of cost-effective, easier and broader application of this effective therapy. Autologous engineered-T cell manufacturing requires a time-consuming process with long vein-to-vein times, while sometimes the production can be unsuccessful (9). Patient-derived products are highly variable, depending on the type and stage of the disease, previous therapies and immune cell composition leading to variable clinical outcomes (10). Allogeneic T cells from healthy donors could provide a solution to several of the aforementioned limitations and are being currently clinically tested as an alternative source of CAR T cells (11). Obviously, substantial genomic editing of the T cell receptor (TCR) and HLA genes is essential for the use of allogeneic cells in order to avoid graft-versus-host reactions and limit graft rejection. However, genetic engineering of primary autologous or allogeneic T cells in a multiplex manner is very challenging (12), as it currently results in a) reduced production yield; b) genotoxicity due to undesired off-target effects and gene translocations; c) an exhausted T cell phenotype and product due to the requirement of extended ex vivo expansion.

To overcome these limitations, the use of induced pluripotent stem cells (iPSCs) has been proposed as an off-the-shelf source of therapeutic T cells (13). iPSCs can theoretically grow infinitely, are easy to genetically manipulate, and can differentiate into different types of immune cells, including T cells. Thus, iPSC have the potential to serve as an unlimited source of T (iT) and CAR T (iCAR T) cells. In contrast to primary T cells, genetic engineering of iPSCs results in fully modified clonal lines, which could be extensively evaluated resulting in a stable safe source.

Most research-grade T cell-development protocols from iPSC have limited translational potential since they include non-compliant good manufacturing practice (GMP) components, making them incompatible for clinical use due to potential xenogeneic immune reactions. Translating a research protocol into a clinically relevant production process is a critical step in the development of new therapies and interventions. Recent efforts are focusing on the development of scalable and good manufacturing practice compliant protocols with serum-free, xeno-free, and feeder-free procedures.

Here, we aim to provide an overview of the research progress of the last decade regarding the effective generation of functional iT and iCAR T cells from iPSC, while highlighting the future directions essential for translational and clinical development in this field. The production of iT cells rely on a complex, orchestrated and highly regulated differentiation process divided in three pivotal stages: the establishment of a master iPSC clone, the progression through hematopoietic differentiation and the subsequent specification of T cells. We will revisit the available research protocols, paying special attention to their alignment with GMP standards, scalability considerations, and their potential for clinical application. Additionally, the integration of safety measures and quality controls for clinical application will be explored, as these facets constitute imperative prerequisites for the eventual clinical deployment of iT and iCAR T cells.

## 2 Good manufacturing procedures in a nutshell

The successful translation of a research protocol into a clinically relevant production process is a critical step in the development of new therapies and interventions. Current research-grade protocols for T cell development from iPSC have demonstrated efficient iCAR T cell production; however, there are still several challenges that are being preclinical addressed, while the first clinical application of iCAR T cells is ongoing (NCT04629729) (14). These efforts to expand the clinical use of iCAR T cells are currently focused on achieving efficient GMP-compliant iPSC generation, cultivation, genetic modification and differentiation towards mature T cells. The labor-intensive nature of preparing the product for clinical use includes the development of GMP-compliant manufacturing practices, standardized procedures, scalability considerations and adherence to other directives and regulations (15).

All reagents, raw materials and disposables must meet the highest available quality standards, preferably manufactured under GMP guidelines. Rigorous quality control systems and standard procedures extend not only to reagents but also to processes themselves, ensuring both the quality and the consistency of the protocols used during manufacturing. Certified and qualified materials and equipment, reliable suppliers, detailed SOPs (standard Operational Procedures) and process validation steps are incorporated in the manufacturing and every procedure is then performed in fully equipped dedicated cleanrooms (16).

In the context of research-grade culture and differentiation, available protocols, that usually involve co-culture with animal-derived components, raise regulatory concerns related to variability between batches, scalability and safety due to potential xenogeneic immune reactions. Therefore, a shift towards the use of human or chemically defined components in the culture, genetic modification and preservation of cells is essential (16). This transition sets the stage for future endeavors towards up-scaling to bioreactor technology and therefore industrial scale production. We note, however, that there might be differences between regulatory regions of the world regarding the obligatory requirements of the use of xenogeneic materials (17). If no other compliant alternatives is available, and sufficient control (testing and sensitivity) can be shown, the use of a non-compliant material could be defended at the competent authority.

Prioritizing a translational approach from early preclinical stages and addressing the necessary steps required for GMP compliance will enhance the prospects of successfully translating research outcomes into clinical applications, facilitating a smoother transition and precluding potential bottlenecks. Given the absence of a standardized method for generating iT cells, substantial efforts are still needed to establish robust, reliable and GMP-compliant manufacturing protocols. Moreover, these protocols should allow a more efficient, clinical-grade production, emphasizing the generation of the T cell subtype with the desired functionality (cytotoxic CD8αβ cells but also helper or regulatory CD4 cells) (18, 19). A pivotal aspect of this effort involves the establishment of relevant quality control and bioassay tests tailored to the specific medicinal product, as will be discussed further.

## 3 Generation of engineered iPSC clones

**Figure 1:**
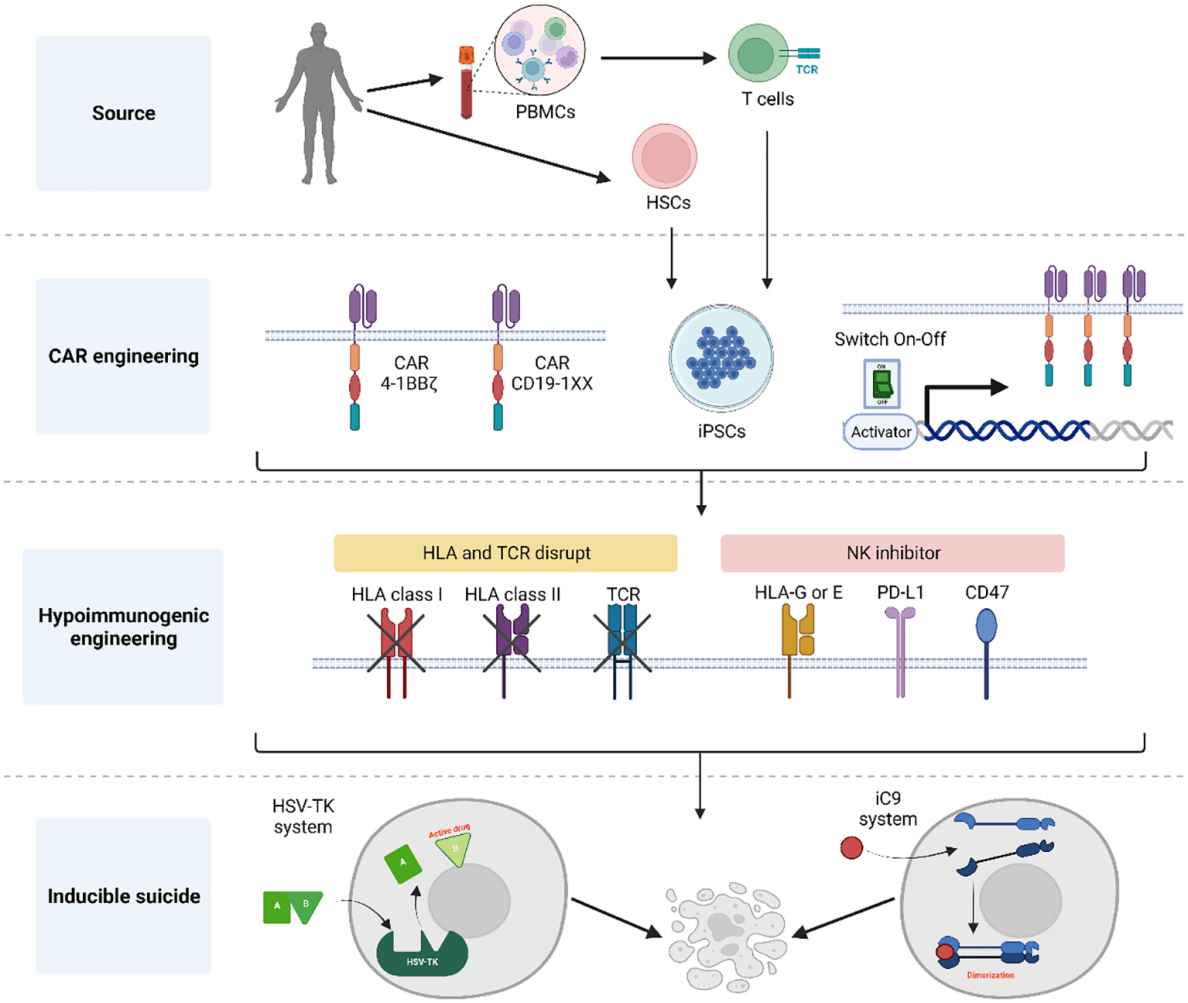
Summary of general aspects for the generation of CAR-engineered iPSC clones. Initially, either CD34+ cells or T cells obtained from donors are reprogrammed into hiPSCs using a genomic integration-free method. Following thorough characterization of pluripotent cells, these hiPSCs are engineered to express a CAR for targeted tumor cell killing. Notably, the CAR construct incorporates elements to control its expression and prevent the effect of tonic signals during T cell development. To achieve a universal T cell product, the hiPSC-derived CAR T cells undergo HLA and TCR elimination with the addition of NK inhibitors to protect against host NK rejection. Additionally, an inducible suicide gene system is introduced into the CAR hiPSCs, serving as a fail-safe mechanism the event of unforeseen complications following T cell infusion. Created with BioRender.com.

iPSCs can theoretically support endless genome editing to accommodate desired characteristics of their lymphoid derivatives, such as tumor-specificity, enhanced function and histocompatibility. Different genetic modifications may also have an impact on the quality and yield of iT and iCAR T cell products. The accommodation of as many as possible optimized immunotherapeutic properties in the iCAR T cells, requires the facilitation and flexibility of gene editing processes. What are key aspects to consider when developing a CAR-engineered iPSC master clone? (See summary in **Figure 1**).

### 3.1 Reprogramming process and donor cell of origin.

The first attempts to generate hiPSCs succeeded by using integrated retroviruses to express the specific genes encoding for the reprogramming transcription factors (Oct4, Sox2, Klf4, and c-Myc) (20, 21). Alternative methods for generating hiPSCs without integrating foreign genetic material have been explored to address these issues including using Sendai viruses (22), episomal vectors (23, 24), synthetic modified mRNA (25), minicircle DNAs (26), microRNAs (27), and proteins (28). While integration-free reprogramming methods have different reprogramming efficiencies, they hold the most promise for developing clinical products due to the lower risk of insertional mutagenesis.

Interestingly, there is much evidence that hiPSCs retain epigenetic (29, 30) and transcriptomic (31) characteristics resembling their original cell type, a phenomenon known as somatic memory. Somatic memory could influence the differentiation potential of hiPSCs, causing them to preferentially differentiate into the cell type of origin, sometimes at the expense of other lineages. Specifically, hiPSCs derived from umbilical cord blood cells exhibit enhanced efficiency in differentiating into the hematopoietic lineage compared to hiPSCs derived from fibroblast (31, 32) and keratinocyte (30), despite showing comparable pluripotent characteristics at their undifferentiated state Notably, unlike murine iPSCs, where somatic memory fades after 10 passages, the observed differences in differentiation potential among hiPSCs remained consistent even after extended culture of up to 20 passages (29, 33),. Up to date, T cells have been generated from T cell-derived iPSC (TiPSC) (34, 35) as well as from non-T cell-derived iPSC (other hematopoietic cell or fibroblast) (36–39), while no detailed studies have been performed on the impact of the cell of origin on the functional properties of the generated iT cells.

### 3.2 Universally applicable iPSC clones

In the context of universally available products, various strategies have been suggested in order to endow iT and iCAR T cells with reduced immunogenicity and alloreactivity. Mismatched MHC class I on adoptively transferred T cells triggers host-versus-graft (HvG) responses from endogenous lymphocytes, limiting the survival and persistence of therapeutic donor cells (40). The disruption of *β2m*, the gene encoding for β2-microglobulin, is the most common strategy for partially overcoming HLA matching barriers (41, 42). B2M monomers combine with major histocompatibility class I (MHC I) molecules, which are present on the surface of all nucleated cells, including T cells (43). However, loss of HLA class I expression results in a “missing-self” response, in which cells lack an essential inhibitory ligand, making them susceptible to attacks by natural killer (NK) cells (44, 45). To address this issue, several additional strategies have been explored. One approach involves the combination of *β2m* knock-out with the introduction of HLA-E over-expression. HLA-E binds to the CD94/NKG2A receptor on NK cells (46, 47) and protects edited cells from NK-mediated killing (48). However, this approach does not address the case of NK cells lacking the NKG2A receptor. Another approach to impair the NK cell response against HLA class I-negative cellular products is the overexpression of CD47, a molecule that serves as a “don’t eat me” signal (49). To enhance immunological tolerance, a different strategy entails deleting both *β2m* (HLA class I) and *CIITA* (HLA class II) genes in iPSCs while introducing HLA-G, CD47, and PD-L1 (50). This strategy is based on the knowledge that HLA-G1 can suppress KIR2D^+^ NK cell populations (51–53) and the well-established immune checkpoint inhibitor PD-L1 inhibits T-cell activation (54, 55). Another group developed selectively deleted HLA-A, HLA-B, and HLA-class II but retained HLA-C, a non-canonical HLA molecule (56, 57), that is expected to inhibit the activation of NK cells (58). In addition, a recent study reported the generation of human iT cells that can evade NKG2A^+^ and DNAM-1^+^ NK cell recognition, by knocking out the NK cell activating ligand PVR as well as both *β2m* and *CIITA*, while simultaneously introducing HLA-E through transduction in hiPSCs (59).

Even when using hypoimmunogenic iT cells lacking expression of HLA molecules, Graft-versis-host disease (GvHD) can still pose a significant challenge due to the presence of T cell receptors (TCRs), possibly leading to the alloreactivity of donor iT cells. Genetic disruption of the TRAC locus to eliminate αβTCR surface expression has become a widely used gene editing method to prevent GvHD in allogeneic CAR T cells (60–62). One strategy, for instance, involves integrating the CAR transgene into the TRAC locus, resulting in TCRless iPSC-derived CAR T cells with reduced risk of triggering GvHD (14, 63).

### 3.3 Expression of the CAR transgenes

Endowing human iT cells with tumor specific functions has been achieved by the expression of a CAR (34). Introduction of the CAR transgene in order to generate iCAR T cells can be performed either by genetic engineering in the iPSC level (34, 64) or at the stage of the already generated iT cells (65). Generation of CAR-engineered iPSC clones has the advantage of requiring only a single genetic engineering step and providing after differentiation to the lymphoid lineage a more uniform iCAR T cell product. However, the CAR engineering strategies employed can impact the lymphoid lineage commitment during differentiation. Premature and constitutive expression of a CAR during iPSC-to-T cell development reduces NOTCH1 (Notch receptor 1) expression and disrupts the normal regulation of downstream genes,leading to lineage skewing of iCAR T cells to innate/γδTCR-like CD8αα^+^ T cell features (34, 66), and expression of NK cell surface markers (34, 38, 66). To partially restore the conventional CD8αβ phenotype (63), it is crucial to regulate CAR signal strength and the timing of expression. Additionally, the choice of different CAR designs plays a significant role in influencing the iT phenotype since 1BBζ-based constructs allow the development of CD8αβ iT cells in contrast to CD28ζ-based CARs (67, 68).

### 3.4 Gene editing

Besides viral delivery methods, the use of gene editing has emerged as the most efficient strategy to introduce genetic modifications into hiPSC, including the overexpression of transgenes or the silencing/knockout of specific genes. The initial nuclease families used were: meganucleases, zinc finger nucleases (ZFNs) and transcription activator-like effector nucleases (TALENs). The emergence of the Clustered Regularly Interspaced Short Palindromic Repeats (CRISPR)-associated nucleases (Cas) system has revolutionized genome editing due to its simplicity and cost-effectiveness. CRISPR/Cas9 has been used to edit *β2m*, *CIITA, HLA* and *TRAC* genes in iPSC (49, 59, 69–72). Furthermore, CRISPR/Cas9 can facilitate multiplex genome editing, allowing for the simultaneous modification of multiple genes through the use of multiple guide RNAs (56, 57). In addition, the CRISPR/Cas9 system has been harnessed for precise gene insertion at specific genomic locations through homologous recombination. For example, it has been utilized to insert a CAR into the TRAC locus, enabling the creation of TCR-less CAR T cells derived from hiPSC (63) and insert NK inhibitor molecules into the safe harbor site AAVS1 locus (50). While CRISPR/Cas9 genome editing avoids random integration events, it carries the risk of adverse genomic events by introducing double-strand breaks at off-target sites (73, 74), which can lead to frameshift mutations, chromosomal translocations, and complex rearrangements within edited cells (75–77). Newer generations of high fidelity Cas9 nucleases have been developed with reduced off-target activity (Cas12a) (78). Nickases, which are modified Cas9 nucleases containing only one functional domain to generate a DNA single-strand break, can be combined with base editors (BE) to induce specific mutations. Finally, the prime editing method involves a nickase fused to a reverse transcriptase complexed with a prime editing guide RNA and can generate targeted insertions, deletions or base substitutions (79).

### 3.5 Safeguard systems

The use of hiPSC as a cellular source for therapeutic clinical applications is accompanied by safety concerns regarding the potential tumorigenicity of their derivatives as well as contamination of the final product with residual undifferentiated iPSC that could potentially lead to the uncontrollable formation of a teratoma. Additionally, strategic engineering to confer hypoimmunogenic traits to these cells, including the absence of MHCs and the expression of CD47 and PD-1, could make them resistant to immune surveillance. To address the risks associated with tumorigenicity, inducible suicide gene systems can be used as a safeguard system. The Herpes simplex virus-thymidine kinase (HSV-TK) system (80, 81), when combined with ganciclovir, effectively eliminates tumorigenic cells in murine iPSCs (82) and human iPSCs (83). However, HSV-TK is a viral protein which can trigger an immune response against the transplanted cells. Moreover the HSV-TK system entails a relatively slow process of cell killing, and is often incomplete due to its inability to target slowly growing cells effectively (84, 85). In contrast, the inducible caspase-9 (iC9) protein, a fusion protein of human caspase-9 and a modified FK506-binding protein, can operate independently of the cell cycle, enabling the rapid initiation of apoptosis of transduced cells within a few hours. The iC9 system can induce of iC9-expressing TiPSC and iCAR T cells *in vitro* and *in vivo* without alteration in pluripotency and T cell differentiation potential of TiPSC (86). Genome editing can further refine this system by targeting integration of the iCasp9 cassette to a safe-harbor locus.

## 4 Considerations for cGMP-generation of engineered iPSC clones

**Figure 2:**
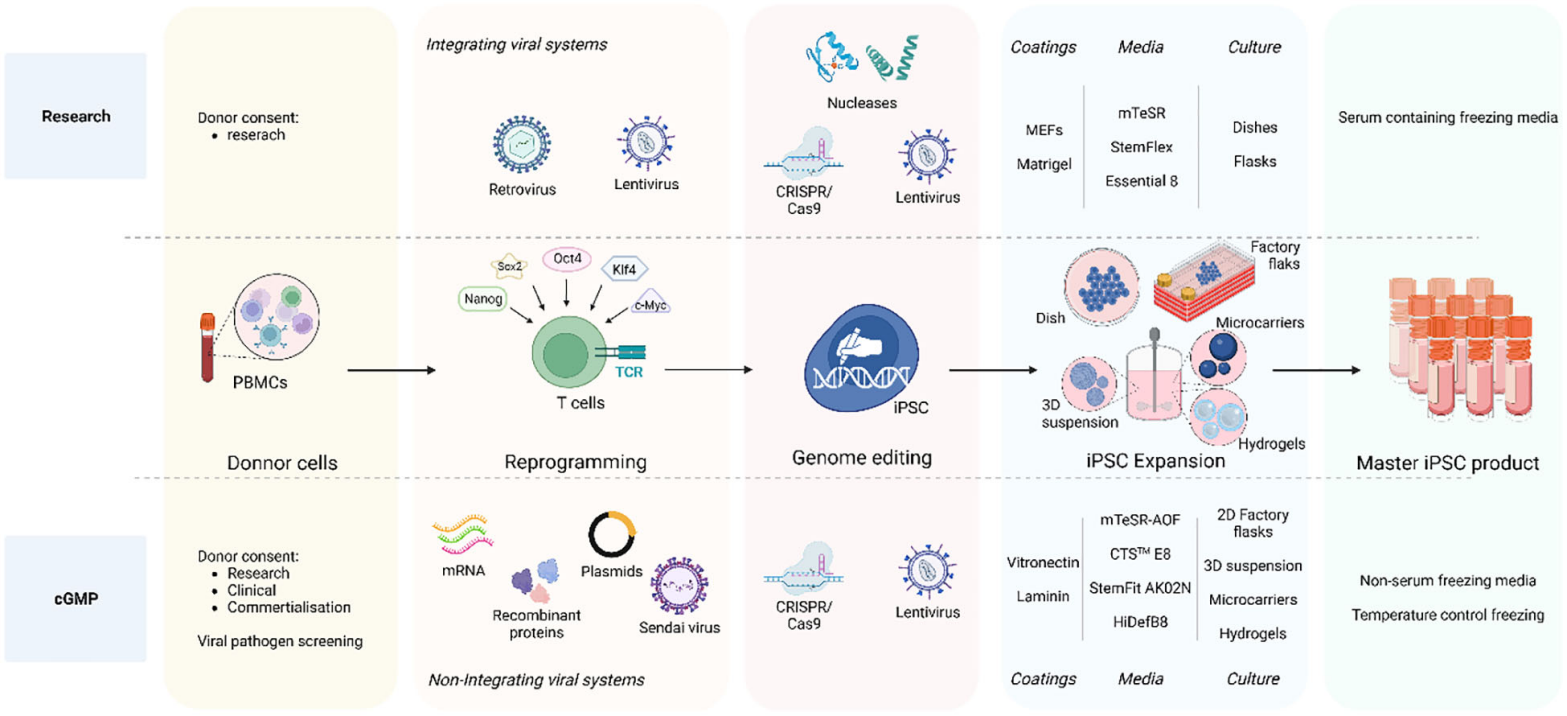
iPSC clone development: research vs cGMP methods. The overall goal is to ensure that the entire process, from donor acquisition to iPSC expansion and culture, adheres to strict quality and safety GMP standards essentials for clinical applications and therapies. The process initiates with the donor cell acquisition adhering to ethical guidelines and obtaining legally valid informed consent. The comprehensive informed consent covers various aspects including potential applications in research and therapy, considerations for commercial use, disclosure of incidental findings, and intervention-specific issues. Non-integrative methods are employed for clinical-grade iPSCs reprograming such as recombinant proteins, DNA plasmids, Sendai virus, and mRNA-based approaches to minimize the risk of genetic alterations as compared to integrating viral systems also used in research. A crucial distinction between research-graded iPSCs and cGMP-compatible iPSCs lies in the iPSC expansion step. Research-grade iPSCs can be cultured on MEFs or Matrigel with animal protein-containing medium, whereas cGMP iPSC culture must reach a defined animal origin-free, serum-free, xeno-free environment. This requirement extends to the components of reagents for iPSC dissociation and cryopreservation media. Furthermore, clinical-grade iPSCs undergo development in a scaled-up culture system using an automated, closed system designed for industrial use. To address these challenges, cGMP iPSCs can be cultivated in 2D factory flasks and in suspension within bioreactors, employing diverse methods such as 3D iPSC spheroids, culturing onto microcarriers, and encapsulation in hydrogels. Created with BioRender.com.

The efforts to make full cGMP iPSC lines (**Figure 2**) starts by obtaining the donor cells, while following the appropriate guidelines including giving written and legally valid informed consent. The informed consent should include terms for potential research and therapeutic uses, potential for commercial application, disclosure of incidental findings and issues specific to the intervention type (16, 17, 87). In order to overcome the concerns of insertional mutagenesis of viral vectors, several non-integrating reprogramming methods have been developed. Recombinant proteins (88), DNA plasmids (23, 89), Sendai virus (90–93) or mRNA-based methods (25, 94) are all non-integrative technologies used to transiently express transcription factors needed to induce cell reprogramming. Overall, although these methods show lower reprograming efficiency than integrating methods and inconsistency the reprograming frequencies are still sufficient to recover iPSC clones when starting from sufficient cell numbers. mRNA transfer has been the most efficient non-integrating reprogramming technology so far, but it is considered a laborious technique and the generated iPSC show extremely low rates of aneuploidy and karyotype abnormalities (95, 96). Nevertheless, comparison of the reprogramming methods within the same starting material, culture time and conditions and comparison to the parental cells is still missing to properly assess differences in efficiency and potential genomic instabilities.

Focus on optimizing ingredients, quality and consistency of matrices and media have reach considerable development towards a defined animal origin-free, serum-free, xeno-free iPSC culture environment certifiable under cGMP. To ensure suitability for clinical grade protocols several synthesized human recombinant extracellular matrix (ECM) proteins or peptides have been developed for iPSC maintenance. Laminin, a major ECM protein during embryogenesis, has proven an efficient adhesion, survival and iPSC self-renewal (97–99) and has become popular as a natural scaffold to transition from research to clinical trials due to its capacity for cell expansion (100) in cost-effective and time-efficient method (101). Other ECM substrates like fibronectin or vitronectin as well as synthetic defined substrate based coatings (ex. Synthemax II-SC, CellStart) have been also used (102).

The culture medium used is another critical component as it provides the necessary growth factors and optimal microenvironment for iPSC maintenance. Chemically defined, animal origin-free culture media (ex. mTeSR-AOF (103–105), StemFit AK02N (65, 106), Essential 8 (E8) (102, 107) (108), HIDEF-B8 (109) as well as cryopreservation media options are commercially available. As enzymatic passaging of iPSC is associated with increased genomic instability (110), chemically defined, enzyme-free dissociation methods (EDTA based) were developed to circumvent this problem (111). Selection of the optimal cGMP conditions should be based on keeping the balance between iPSC expansion, preservation of pluripotency and genomic stability (112).

To satisfy the increasing clinical requirement of iPSCs the potential of scaling up iPSC culture several automated platforms are available to provide automated, closed system and customizable settings for coating, cell seeding, media feeding and cell harvesting of adherent cells such as the CliniMACS Prodigy Platform (Milteny) or the Quantum Cell Expansion System (100). In this later, capillary tubes are coated with matrix and then loaded with iPSCs providing a very large surface area for cells to attach to, while keeping a small physical footprint. However, in this system, cell growth is challenging to monitor (113) and the expansion of iPSC culture still remains modest for industrial use. To overcome these issues, various possibilities to scale up iPSC culture in suspension in bioreactors exist including self-assembled 3D spheroid aggregate (114–120) culture and cultivated cells in microcarriers or hydrogels. Cells can be cultured on microcarriers, increasing the cell surface while maintaining reproducible suspension cultures in closed systems (121–124). Several types of microcarriers are available with different coatings, cell attachment properties and sizes, that will provide different cell expansion and pluripotency maintenance while maintaining normal genomic stability (125–127). Microencapsulation of iPSC cells with hydrogels, like alginate, is also a potent option for successful up-scaled iPSC maintenance and expansion (128–131). However, cost effectiveness of some of these methods still needs to be considered.

Finally, quality control testing and release criteria need to be established to ensure product sterility, cell identity and characterization and genetic stability of the iPSC master product (**Table 1**). To this end, the international stem cell banking initiative (16) and the Global Alliance for iPSC therapies (132) currently provide general guidance regarding clinical-grade iPSC standards. Since procedures to obtain master iPSC lines are not always performed under full cGMP and the cell product itself cannot be sterilized, donors and the resulting donor cells should be screened with rigorous viral (human immunodeficiency virus HIV, hepatitis B virus, and hepatitis C virus), bacterial, fungi, mycoplasma and endotoxin sterility tests. Moreover, clinical grade iPSC cells need to be fully characterized to confirm cell identity, genomic integrity, pluripotency, purity and potency as well as to manage the risk associated with the presence of atypical or spontaneously differentiating cells. Cell reprograming efficiency and purity of the cell line is widely evaluated by flow cytometry and immunostaining, analyzing expression of commonly used pluripotency stem cell markers like Oct4, Nanog, Sox2, TRA-1-60, TRA-1-81, SSEA-3 and SSEA-4. A minimum of two positive markers from this panel is mandatory, combining at least one intracellular (Oct4, Nanog, Sox2) and one extracellular marker (TRA-1-60, TRA-1-81, SSEA-3 and SSEA-4. Positive expression of these markers should be consistent and homogenous in the population analyzed with over 70% of positive cells (133, 134). Based on the description reported by Yu et al. (2007) (21), iPSC culture should maintain their typical morphology with compact rounded colonies and smooth edges, and a high nucleus-cytoplasm ratio. Genome integrity of the iPSC line is evaluated by single tandem repeat (STR) genotyping to confirm a matching identity between the source cells and the reprogramed cell line (102, 134). For gene-edited iPSC lines, assessment of the guide RNA quality, presence of the wildtype sequence, proof of any off-target effect and sequence analysis of the target pre and post editing needs to be provided. Lastly, testing for the presence of reprogramming vectors is mandatory to confirm clearance or silencing of these vectors. Clearance to an acceptable threshold of <1 plasmid copy per 100 cells must be demonstrated (134). Tests to currently show clearance of Sendai virus and mRNA methods are being developed. Potency of the generated cells is confirmed by carefully analyzing their differentiation potential into all three germ layers (ectoderm, endoderm and mesoderm) (100, 133, 134). The most widely accepted and available of these are Pluritest™ (135) and hPSC ScoreCard™ (136). Finally, iPSC are known to be prone to genomic instability which raises potential hazards around cell transformation and risk for causing malignancies in patients. Some consensus and guidelines are slowly emerging to assure genome integrity and stability of iPSC (137, 138). Karyotyping, using G-banding, has been considered the gold standard method to report karyological analysis of iPSC. A universal clinical standard recommended is a 20-metaphase karyotypic analysis with 95% certainty of diploidy and is widely accepted by regulators worldwide. Newer and more accessible technologies are being developed as KaryoLite BoBs^®^ and Single nucleotide polymorphism (SNP) arrays although each have significant differences in sensitivity, resolution and each has limitations for routine QC in terms of cost and time (133). SNP array offers higher resolution, enable to detect subchromosomal changes and single point mutations, deletions or duplications. SNP is recommended for information only, while karyotyping is a mandatory control. Extra whole genome analysis can also voluntarily be performed. Online tools such as the Decipher interface or the Variant Effect Predictor can help to identify genomic abnormalities and predict the possible effects (138).

**Table 1:** Quality control assessment of iPSCs clones.

	Quality attribute	Assay	Criteria
**Sterility**	Viral pathogen (HIV, HBV, HCV)	qPCR	Not detectable
	Bacterial	qPCR/broth	
	Fungi		
	Mycoplasma		
**Cell Characterization**	Purity	Flow cytometry	Above 70% positive pluripotency stem cell markers
		Immunostaining	
	Viability/Morphology	Visual assessment	
	Cell identity	STR genotyping	Matching identity
		Virus/Plasmid clearance by PCR	<1 plasmid per 100 cells
	Potency	Teratoma	Trilineage differentiation capacity _ Upregulation trilineage genes
		Spontaneous or directed differentiation _ PCR	
**Genome integrity & stability**	Karyotyping	G-banding	
		SNP	
		Whole genome	

The combination of the different tests and criteria should provide enough information to determine the quality and safety of the iPSC line. Although specific assays and standards are not yet defined, consensus on specific parameters and guidelines on essential information needed is being build up in the area to ensure successful clinical grade iPSC application.

## 5 Differentiation of iPSC towards hematopoietic cells

In order to develop protocols and methods to generate cells of the hematopoietic lineage, including T lymphocytes, from iPSC one should take into account the physiologic developmental processes. Hematopoietic stem and progenitor cell (HSPC) development occurs in three sequential waves of cells characterized by distinct locations, timing, and differentiation potential (139, 140). The initial wave, referred to as “primitive hematopoiesis,” occurs at the yolk sac and is responsible for generating various blood cells that together establish the embryo’s blood circulation and initial immune system (141). The second, pro-definitive hematopoiesis, originates in yolk sac and provides erythro-myeloid progenitors and lympho-myeloid progenitors that migrate across the embryo, supplying blood cells until the time of birth (142). The third wave, termed “definitive hematopoiesis” emerges within the embryo from hemogenic endothelial cells in the dorsal aorta of the aorta-gonad-mesonephros region. Unlike the previous waves, this wave is capable of giving rise to the first true HSPCs that exhibit robust long-term self-renewal capabilities and have the potential to differentiate into all hematopoietic lineages, including mature erythrocytes switching from fetal to adult globin, as well as all lymphoid lineages, including B cells and T cells. Definitive HSPCs produced during this stage initially localize in the fetal liver and subsequently colonize in the bone marrow, where they facilitate lifelong hematopoiesis (143, 144).

The initial step to successfully generate definitive HSPCs *in vitro* involves inducing mesoderm lineage through primitive streak by specific signaling pathways like bone morphogenetic protein (BMP), activin/nodal and Wingless/Integrated (Wnt) pathway (145). In the early stages of mesodermal specification, the expression of Brachyury (T), followed by the expression of KDR kinase insert domain receptor (KDR), also referred to as vascular endothelial growth factor receptor 2 (VEGFR2), is pivotal for further differentiation (146). VEGFR guides mesoderm cells with KDR to transition into the endothelial cell stage. Within this endothelial population, a distinct cell type known as hemogenic endothelium (HE) exists with potential to give rise to hematopoietic lineage cells. The HE exhibit markers typical of endothelial cells, including platelet endothelial cell adhesion molecule PECAM-1 (CD31), VE-cadherin (CD144), and hematopoietic lineage progenitor marker CD34 (147, 148). In the final step, a significant morphological shift occurs as these cells detach from neighboring endothelial cells, forming round floating clusters of HSPCs for definitive hematopoiesis. This cellular transition, termed the endothelial-to-hematopoietic transition, occurs after hematopoietic transcription factors such as Runx1, Gata2, and Sox17 are upregulated within a specific subgroup of HE cells (149). Consequently, these newly generated HSPCs express not only CD34 but also CD43, a pan-hematopoietic marker that distinguishes them from endothelial cells (150).

Various methods have been established to generate HSCs from hiPSCs and hESCs *in vitro*, aiming to closely replicate the key features of definitive hematopoiesis as described above. The hematopoietic differentiation *in vitro* can occur through three distinct approaches. Firstly, it can be conducted via co-culturing with murine stromal cells such as S17, MS5, C3H101/2 and OP9 (151–154). Notably, the OP9 stromal cell line was initially derived from a newborn mouse carrying a mutation in the macrophage colony-stimulating factor (M-CSF) gene (155). Due to this mutation, OP9 stromal cells are unable to produce functional M-CSF, which in turn prevents them from supporting the differentiation of macrophages. Nevertheless, the OP9 cell line has emerged as the most widely used stromal cell line to induce hematopoietic differentiation of hPSCs. The efficiency of OP9 co-culture induction can be improved by various combinations of factors such as stem cell factor (SCF), BMP4 and FMS-related tyrosine kinase 3 ligand (FLT3L). The studies have provided evidence of the efficiency of murine stromal cell co-cultures in generating definitive hematopoietic progenitors with lymphoid potential (153, 155) and long‐term engraftment potential (156). However, the use of mouse cells poses compatibility challenges for clinical product development due to concerns about xenogeneic antigen contamination.

A second approach is the spontaneously formed 3D organoids named embryoid bodies (EBs). EBs faithfully replicate the spatial organization observed in embryo development, closely mimicking the multicellular arrangement found in actual human organs. This 3D structure promotes cell-to-cell interactions, facilitates cell communication, and allows for the exchange of substances. Consequently, EB systems have been widely utilized to generate specific cell types, including hematopoietic cell lineage, by employing combinations of inhibitors, small molecules, and other growth factors. Sturgeon et al. successfully guided hiPSC to differentiate into definitive CD34^+^ HSPCs by inhibiting activin-nodal signaling through SB431542 or activating the Wnt signaling using CHIR99021 during the EB formation in serum-free condition. Remarkably, the CD34^+^ cells produced through this method demonstrated the ability to differentiate into erythrocytes expressing adult globin and lymphoid cells (157). Furthermore, when combined with genetic modification involving the overexpression of specific transcription factors, the EB method effectively generated HSPCs with engraftment potential in a mouse model (158–160).

Spherical formation of EBs presents challenges due to significant heterogeneity of differentiated cells and difficulty in monitoring hematopoietic processes within them. As a result, 2D monolayer feeder-free method for inducing hematopoietic differentiation *in vitro* has been developed iPSCs are seeded onto a surface coated with extracellular matrices and subsequently, different combinations of cytokines and small molecules are introduced at specific stages during the culture to lead the 3-stage differentiation process (35, 161–165). These refinements have simplified the monolayer method into a cost-effective and straightforward strategy for generating HSPCs suitable for clinical applications.

## 6 *In vitro* T lymphoid development

Physiological T-cell development takes place within the thymus, where bone marrow-derived hematopoietic progenitors migrate through the bloodstream, commit to the T-cell lineage and undergo further maturation to become functional T lymphocytes (166). This commitment and maturation process is largely driven by the Notch signal transduction pathway, which plays a central role in initiating a T-cell gene program in these cells upon their arrival in the thymus (167, 168). Upon their entry into the thymus, these progenitors undergo a commitment to become early thymic progenitors (ETP). Following this initial commitment, ETPs embark on a journey through a series of differentiation stages, culminating in the formation of mature T cells. These stages include the development of immature CD4/CD8 double-negative thymocytes, immature CD4 single-positive thymocytes (ISP cells), and immature CD4/CD8 double-positive thymocytes (DP cells). Following a series of positive and negative selection processes, DP cells undergo further maturation, giving rise to two distinct subpopulations: naïve mature CD4 single-positive (CD4SP) thymocytes and CD8 single-positive (CD8SP) thymocytes (169).

The recognition of Notch signaling as a critical regulator of T cell commitment has resulted in significant advancements in methods for generating T cells *in vitro*. Initially, researchers employed an OP9 cell line that had been genetically modified to overexpress Notch Delta-like ligand 1 (DL-1). Similar to thymic stromal cells, this modified cell line, referred to as OP9-DL1, was employed to establish a co-culture system, which served as the starting point for initiating T cell development *in vitro*, including interleukin 7 (IL-7), stromal cell-derived factor 1 (SDF-1), and stem cell factor (SCF). The OP9-DL1 co-culture system enables the generation of functional CD3^+^ TCR^+^ iT cells *in vitro* (170). Interestingly, in *in vivo* models, the targeted knockout of Dll1 in the thymus does not affect T cell development (171). In contrast, Dll4 is an indispensable Notch1 ligand in T cell development (172). The key distinction between Dll1 and Dll4 is that Dll4 exhibits a higher binding capacity to the Notch1 receptor. Therefore, Dll4 appears to be more effective than Dll1 in activating Notch and initiating a T cell lineage at the early stages of T cell development (173). In the context of T lineage development from hiPSCs, OP9-DL1 as well as OP9-DL4 successfully supported the development of DP cells from hiPSCs with germline TCR genes (37, 174, 175). However, it’s worth noting that when TiPSC are used as a source on the OP9-DL1 system, there is no development of DP thymocytes and the regenerated iT cells do not express surface markers of cellular maturity such as CD5 and the CD8αβ dimer, while expressing CD8αα and CD56, indicative of an innate-like phenotype (34, 66, 90, 176). The pre-mature expression of the pre-rearranged TCRα genes during *in vitro* differentiation is responsible for this lineage skewing and use of stronger Notch activation though OP9-DL4 can restore the progress to the DP stage (63). Furthermore, this system has been used to generate functional iCAR T cells, exhibiting specific killing potential in *in vitro* and *in vivo* mouse models (34, 38, 66, 177). Similar to early TCRα expression, CAR signaling during differentiation hampers the development of DP and mature single positive CD8αβ iCAR T cells (63, 67, 68).

In recent years, feeder-based systems have advanced to mimic better the thymus’s 3D structure, which plays a pivotal role in facilitating positive selection and improving human T cell development (178, 179). The artificial thymic organoid (ATO) platform closely mimics certain aspects of the 3D thymic structure. In this innovative approach, DLL1- or DLL4-expressing MS5 stromal cells are aggregated by centrifugation with mesodermal progenitors from hESC or hiPSC, creating a 3D environment conducive to T cell differentiation (36). The DLL1-expressing MS5 cells in ATO efficiently support the development of anti-CD19 CAR T cells derived from hiPSCs, preserving CAR expression and function. These hiPSC-derived CD19-CAR T cells exhibit comparable specific cytotoxicity, cytokine secretion and efficacy in controlling the progression of CD19^+^ leukemic cells in animal models when compared to PBMC CD19-CAR T cells (64). Interestingly, recent studies have highlighted the ATO system’s ability to induce the differentiation of CD4^+^ iT and iCAR T cells. Nevertheless, the functionality and potential of these CD4^+^ T cells remain subjects requiring further investigation (36, 68).

## 7 Considerations for cGMP differentiation of iPSC to iT cells

**Figure 3:**
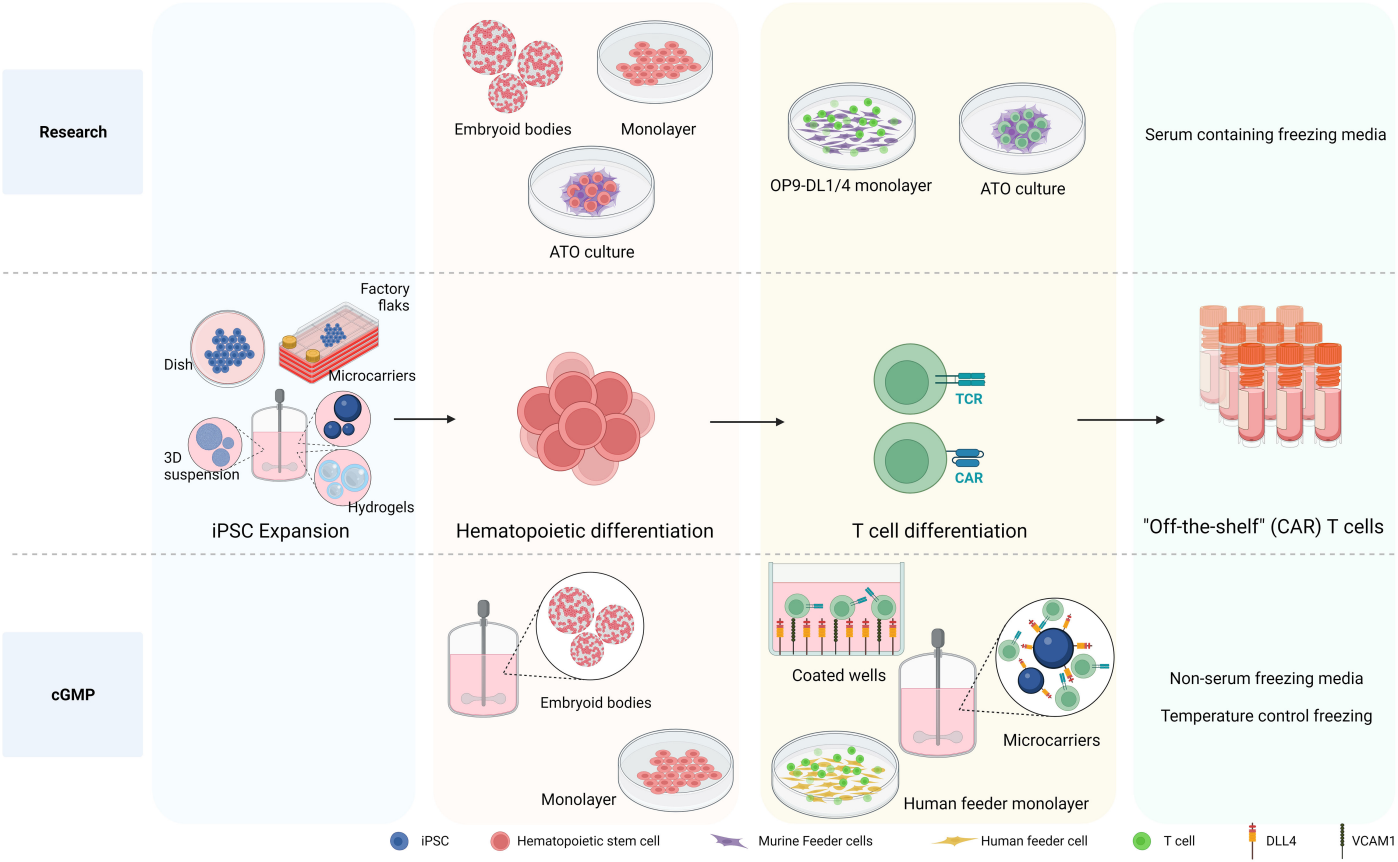
Differentiation processes to generate iPSC-derived CAR T cells. The differentiation processes involved in generating iPSC-derived CAR T cells is depicted, highlighting the transition from research-level methods to those tailored for large production in clinical settings adhering to cGMP standards. At the research level, iPSCs are differentiated into hematopoietic stem and progenitor cells (HSPC) using various methods, including embryoid bodies (EB), monolayer, and artificial thymic organoid (ATO) methods. The necessity of producing HSPCs on a defined, xeno-free, large scale clinical setting makes EB formation in bioreactor systems an attractive approach. While the monolayer differentiation protocol also follows cGMP standards, 2D large scale production might be more cumbersome. For T cell differentiation, murine feeder cells prove to be affective in research settings. Nevertheless, the development of a cGMP-compatible T cell differentiation methods demands the exclusion of xenogeneic antigens, leading to the use of a recombinant DL4 and VCAM1 protein-coated surface or human origin feeder cells. Notably, the protein-coated surface approach arises as an attractive strategy for large scale implementation culturing HSPCs together with coated microbeads in bioreactor systems to give rise to differentiated T cells. Created with BioRender.com.

As mentioned above to date, research grade approaches to differentiate hiPSCs toward HSPCs include either murine bone marrow-derived feeder cells in serum-containing medium (36, 37, 64, 153) or defined conditions with specific growth factors or cytokines via formation of embryoid bodies (EBs) (157, 180) or monolayer system (35, 181, 182) (see **Figure 3**). The first big step forward towards clinical production has been the generation of CD34^+^CD43^−^ HSPC, with a serum-free and stroma cell-free protocol based on embryoid body formation and the use of specific cytokine and growth factor combinations in a stepwise manner by Kennedy et al. (2012) (180). Since then, EBs have been widely used to develop HSPC with diverse differentiation potential towards T cell (34, 63, 181, 183, 184). Although initial EB formation protocols relied on serum-containing media (185), more recent advancements have led to the development of xeno-free and serum-free media for use in these protocols (186–188). Differentiation approaches with defined conditions highly rely on the cytokine/growth factor cocktails used to prime the HSPC towards the preferred lineage, therefore important to be sure that required compounds are GMP grade available. As EBs are 3D aggregates containing a complex cellular composition, the hematopoietic progenitors need to be released from the EBs. Progenitor releasing involves enzymatic dissociation which can be harsh treatments affecting viability and further processes. After cell dissociation, CD34^+^ cells need to be enriched and isolated to proceed for further differentiation in an appropriate cGMP environment.

Possibly simpler, more efficient and cost-effective approaches to generate hematopoietic cells have been established using a feeder-free and serum-free monolayer method to derive HEPs with mature blood cell differentiation potential from iPSC (35). This monolayer method provides large numbers of high purity hematopoietic cells, removing the need of CD34 purification step for both fundamental research and most important for regenerative medicine in a cGMP manner.

As mentioned earlier, conventional methods for further generating iT cells from the iPSC-derived HSPC have used various murine feeder cell lines, presenting significant challenges when designing manufacturing processes compliant with cGMP. Efforts have been made to explore the use of human feeder cells to support T cell development *in vitro*. The results of T cell differentiation in a co-culture of mouse primary fibroblast-DL4 and human HSCs were remarkable. In contrast, human primary fibroblasts expressing DL4 exhibited only minimal capacity to initiate early stages of T-cell development from human HSCs, even when macrophage colony-stimulating factor inhibitors were introduced (189). An alternative approach for establishing a human feeder cell system involved growing primary human fibroblasts and keratinocytes on a 3D scaffold, followed by seeding them with human CD34^+^ cells (190). This method holds promise for producing clinical-grade mature T cells in a laboratory setting. The outcomes of this co-culture revealed that fibroblasts and keratinocytes exhibited an increase in the expression of Dll4 and IL-7 during culture on the 3D scaffold, resulting in successful generation of single-positive CD4^+^ and CD8^+^ T cells expressing αβ TCR. However, the reproducibility of these results has not confirmed (191). Using human thymic epithelial cells (TECs) is another option for feeder cells. A previous study revealed that co-culture of cord blood CD34^+^ cells with a human immortalized TEC line overexpressed DL1 significantly facilitated T cell development and proliferation, progressing CD34^+^ cells to the DP stage. However, these differentiated cells did not proceed to the SP stage (192). A recent protocol has emerged involving the formation of thymic organoids by combining hPSC-derived thymic epithelial progenitors (TEPs), HPSCs, and mesenchymal cells. Within the 3D structure, hPSC-derived TEPs undergo further differentiation into TECs, and these cells can support T cell development *in vitro*, as demonstrated by the detection of αβTCR^+^ CD3^+^ cells after 4 weeks (193).

Self-assembled 3D organoids have been employed in hematopoietic and T cell differentiation, aiming to closely mimic the *in vivo* microenvironment compared to traditional 2D cell cultures. However, 3D organoid models are inherently more complex than 2D cell cultures, posing challenges for standardization. Variability in organoid structures and compositions may impact reproducibility. Additionally, establishing and maintaining 3D organoid cultures can be technically demanding. Researchers may encounter difficulties in optimizing culture conditions, maintaining cell viability, and ensuring consistent outcomes. 3D bioprinting emerges as a promising method for modeling T cell development, offering advantages such as precise control over the spatial arrangement of cells and biomaterials and the ability to achieve a high level of complexity. However, compared to self-assembled 3D organoids, scaling up 3D bioprinting cultures for large-scale experiments can be challenging and requires specialized culture media and equipment. The resulting high costs and associated complexity may make this approach less suitable for mass production.

While using human-origin feeder cells for T-cell generation from iPSCs has faced challenges due to disappointing efficiency and difficulties in scaling up the process, a feeder-free system has been developed for generating T cells from iPSCs. Recombinant DL4 alone has proven to be effective in promoting T cell differentiation from hiPSC. When recombinant DL4 is immobilized on a plate, it successfully generates functional antigen-specific CD3^+^ CD8^+^ T cells (65). Subsequent investigations have revealed that combining DL4 and VCAM1, an adhesion molecule for lymphoid cells, into a coating material enhanced the T cell potential of hematopoietic progenitors from hPSCs during the EHT stage, resulting in a substantial increase in the production of mature CD8^+^ T cells (184, 194). Additionally, employing a feeder-free approach, coupled with knocking down EZH1, the definitive hematopoietic fate repressor, yields a higher percentage of CD3^+^ iT cells and an increased number of differentiated iT cells (184). Finally, the scalable bioreactor for large-scale iT cell production method was established by using a DL4-immobilized bead (183, 195).

Nevertheless, it is important to note that despite the success of feeder-free systems in generating functional SP CD8^+^ T cells, there successful generation of SP CD4^+^ T cells with helper functions is lagging. Prior studies induced positive selection *in vitro* using an anti-CD3 antibody to target the TCR complex in the presence of combinations of cytokines (65, 184, 194). Regrettably, these methods were unable to generate functional CD4^+^ T cells from hPSCs. A recent study introduced a novel method for inducing positive selection of iPSC-derived DP cells, by using low concentrations of PMA and ionomycin within the feeder-free system, resulting in the successful generation of SP CD4^+^ T cells. These CD4^+^ T cells can secrete signature cytokines of helper T cells, such as IL-2, IL-4 and TNF-α, upon activation but do not secrete IFN-γ. Furthermore, when these generated CD4^+^ T cells are cultured in a medium supplemented with TGF-β and IL-2, they undergo further differentiation into regulatory CD4^+^ T cells capable of suppressing T cell activation and proliferation *in vitro* (196).

## 8 Scalability of HSPC and iT production

At research level EBs are maintained in static suspension cultures with limited potential for large scale production and control over cell aggregation and EB formation. Large scale EB formation and differentiation can be achieved in hydrodynamic conditions such as rotary orbital culture, rotating cell culture systems or bioreactor systems, which generally improves cell aggregation and a more homogenous EB formation compared to static methods (197, 198). Shaker flasks or roller bottles with constant circular motion provide a simple system for suspension based mixing environment improving the efficiency of EB formation. Although this technology lacks process control and scalability compared to other methods, it is helpful as a first attempt of scaling up while allowing comparison of different experimental parameters as various shakers and bottles can be accommodated on the rotary platform (197). A key parameter to control during dynamic cultures is the shear stress for the cells in culture as it will determine the cell clumping or dissociation of the cells. Rocking motion bioreactor and rotary cell culture systems are low-shear methods that drive continuous mixing and aeration, yielding high quality and yield EB culture. These systems are available as single use disposable bioreactors which make it advantageous to avoid issues around contaminations from reusable systems (197, 199–201). On the other side, spinner flask and vertical-wheel stirred bioreactors provide an attractive simple design, with scalable configuration, easy continuous monitoring and a flexible culture of cells as aggregated or on microcarriers/scaffolds (200, 201). The paddle impeller inside the bioreactor is responsible to continuously mix the medium having an impact on the cell viability and aggregate size of the culture. Cell aggregates like EBs or cells grown in microcarriers are more sensitive to shear stress than single cells. While low rate of stirring results in cell clumping supporting EB cultures, high rates can be harmful for the cells and dissociate (202). Studies to reach the optimal fluid velocity and promote a suitable shear stress is critical for scaling up in these systems.

In contrast to EBs methods, which have a more straightforward adaptation to bioreactors, CD34 differentiation on a monolayer requires attachment of iPSC to a surface. Microcarriers are reported as a suitable biomaterial to culture cells in suspension instead of planar surface. Commercially available microcarriers differ in size, core and coating material, surface charges and porosity resulting in different cell seeding capacity and expansion. Therefore it is important to select the most efficient microcarrier for each large scale application by screening and assessing the seeding, proliferation, differentiation and harvesting efficiency of the cells of interest such as iPSC culture and CD34 differentiation in this case (203).

To our knowledge, only one scalable approach for T cell differentiation using serum and feeder free protocol has been published (183) and patented (195). Streptavidin microbeads are coated with DL4 and cultured in suspension together with the developed hematopoietic progenitors and differentiating T cells. The best cells-to-beads ratio was assessed using G Rex Gas Permeable Rapid Cell Expansion Devices to successfully develop functional CD8αβ T cells. Other automated, closed, flexible integrated cell manufacturing platforms could also be considered, such as the Cocoon^®^ (Lonza) or the CliniMACS Prodigy (Miltenyi), used already for conventional CAR T cell manufacturing, in order to increase control over the specific processes.

## 9 Quality and safety control of the final iPSC-derived T cell products

Ensuring the quality, safety, and potency of the generated iPSC-derived T and CAR T cells is paramount for successful translational and clinical development. Robust release criteria, along with stringent safety and quality control measures, are essential to guarantee the therapeutic effectiveness and minimize potential risks (**Table 2**). Specific guidelines for CAR T cell therapy clinical release were added in 2021 in a revised version of the EMA Guideline on quality, nonclinical, and clinical aspects of medicinal products containing genetically modified cells and released in 2022 in the “Considerations for the Development of Chimeric Antigen Receptor (CAR) T-Cell Therapies” by the FDA. Release criteria for stem cell-based interventions must encompass qualified and validated assays that comprehensively assess various product attributes, including identity, purity, sterility, safety, and potency.

**Table 2:** Quality control and iT and iCAR T cell release criteria.

Category	Quality attribute	Assay	Criteria
**Safety**	Sterility (microbial, virus, fungi)	PCR Broth	Not detectable
	Mycoplasma		
	Endotoxin	LAL assay	Not detectable
	Genotoxicity	RCL/RCR assay VCN PCR	Not detectable < 5
	Biodistribution	PCR/Immaging	
**Cell Identity**	CAR transgene expression	PCR	
		Flow cytometry	
	% CAR cells	Flow cytometry	
**Cell purity**	Viral particles		Negligible
	Residual unmodified cells	Flow cytometry	
	% CD3 T cells		
	Viability		> 70%
	DMSO content		< 1mL/kg/day
**Potency**	Cytotoxicity	Direct assay (CR release, impedance, bioluminescence)	
		Indirect assay (secretion of cytokines, degranulation)	
	Immunophenotyping	Memory, Exhaustion, Senescence profiles	
	Proliferation capacity		

Similarly to the abovementioned considerations for iPSCs maintenance, rigorous safety testing should be implemented for all production phases, encompassing assays to detect microbial and mycoplasma contamination, adventitious agents, replication-competent viruses, and potential genotoxicity. Sterility assessment involves the detection of microbial or fungal growth by turbidity assessment of the samples after incubation, or by newer and faster methods developed based on colorimetric, fluorescent or bioluminescence assays (e.g., BacT/Alert 3D^®^ and BD BACTEC™ systems, Rapid Milliflex^®^ Detection System) (204). PCR-based assays to detect mycoplasma are also being developed detecting conserved sequences like the bacterial 16S rRNA (205). In the case that integrating viral vectors are used for delivering of a transgene (CAR or other) then recommendations for Replication Competent Lentivirus (RCL) or Retrovirus (RCR) testing encompass evaluating all viral vector lots, manufactured cell products, and monitoring patients’ post-infusion (204, 206, 207). Also, the risk of insertional mutagenesis should be considered and factors influencing the risk include vector insertion profile, design with enhancer and promoter sequences, transgene product, and vector copy number (VCN) per transduced cell. Regulatory agencies mandate integration profile characterization to support marketing authorization applications. VCN per transduced cell analysis is a pivotal safety attribute, aiming to strike a balance between safety and efficacy. Typically, maintaining less than five copies per transduced cell is deemed safe, often assessed using quantitative PCR (qPCR) (208). Single-cell analysis, like droplet digital PCR (ddPCR), offers advantages in detecting cell-to-cell variability and identifying clones with higher integration counts, which may pose greater risks (209, 210). Besides, comprehensive genomic distribution and potential adverse effects of introduced genetic alterations is necessary to evaluate the long-term safety of the product. Techniques like next-generation sequencing (NGS) can be employed to analyze the integration profile of introduced transgenes and assess potential genomic alterations (209). Finally, detailed and sensitive biodistribution studies are essential for understanding how cells distribute throughout the body after administration, whether they are injected locally or systemically. Techniques such as qPCR and imaging modalities (such as bioluminescence imaging or positron emission tomography) can provide insights into the spatial distribution and persistence of administered cells over time (209).

Identity testing should include assays to measure the presence of the transgens and immune-phenotyping of specific cellular populations. such as single positive CD4 or CD8 T cells, to confirm the product’s identity (204). Initiatives such as the Euroflow consortium and the Human Immuno-Phenotyping Consortium are working towards developing streamline and standardize immune-phenotyping assays, focusing on standard antibody panels, internal controls and automated analysis strategies (211, 212).

Purity assessment involves quantifying the relative freedom from extraneous materials in the final product, including both process-related and product-related impurities. Quantitative limits of certain impurities coming from media, supplements, antibiotics or reagents in the final product need to be set. Regarding product-related impurities, given the potential heterogeneity of iPSC-derived products, special consideration is given to residual undifferentiated iPSC due to their potential to form teratomas. To identify residual undifferentiated iPSCs cellular marker panels targeting pluripotency-associated markers (such as OCT4, SOX2, NANOG) can be utilized to detect and quantify them within the product (204). Release criteria for conventional CAR T cell products include % CD3 T cells but full characterization of the product is desirable. A minimum of 70% viability of the CAR T cell product is recommended by the FDA (205) and evaluation of bacterial endotoxin level by for example the Limulus Amoebocyte Lysate (LAL) method is mandatory (204). Finally, to consider the cryopreservation of the cell product where DMSO is used as excipient, however it is toxic if high volumes are infused together with the CAR T cell product. A safety limit for infusion was defined by the FDA as 1 mL/kg/day. Precise cell count and flow cytometry analysis of the CAR expression and cell populations in final product permits a precise assessment of the final dose (204).

The allogeneic nature of iPSC-derived products as well as reports on aberrant protein expression by iPSC-derived products dictates for careful assessment of their potential for allo-immunization before clinical application (213). To date, there are no standardized assays for iT and iCAR T products. Tests analogous to those applied in assessing the immunogenicity of conventional CAR T cells can be extended to iCAR T cells. For example, ELISA and flow cytometry have proven effective in detecting existing antibodies specific to CAR proteins or other components from iCAR T cells (214) and standard mixed lymphocyte cultures can reveal cell-mediated reactions. The potential for *de novo* allo-immunization can be tested in non-human primate models (215).

Preclinical studies should not only provide evidence of product safety but also establish proof-of-principle for therapeutic effects. Potency assessment is a critical component to ensure the function and consistency of cellular products, ensuring batch-to-batch uniformity. Customized assays aligned with product mechanisms and attributes are essential. Although standardized assays for CAR T-cells are yet to be widely established, *in vitro* cytotoxicity assays against target cells are commonly employed (204, 207, 216). *In vivo* assays face limitations due to variability of animal models and technical complexities. *In vitro* functional assays, such as cytotoxicity assays, provide insights into the product’s anti-tumor potential. Immunophenotyping, for example expression of phenotypic markers associated with T cell activation, exhaustion, and memory differentiation, could aid potency assessment by correlating phenotypes with efficacy. However, there isn’t a specific immunophenotypic profile currently identified as a direct predictor of CAR T-cell function in a validated quantitative assay. Indirect assays that measure a by-product of the effector–target interaction can also serve as an indicator of potency such as the secretion of cytokines and chemotoxins (e.g., IFN-γ, TNF-a, IL-2, granzyme B) upon effector cell activation (217). Notably, FDA-approved products like Tisagenlecleucel and Axicaptagene Ciloleucel employ IFN-γ secretion as part of their potency assessment in response to CD19-expressing targets (204). However, when using IFN-γ detection via ELISA, it’s important to recognize that this reflects cytokine release from the entire incubated cell population, which could lead to an overestimation of CAR T-cell cytokine secretion (217). For a more targeted analysis, flow cytometry assays can intracellularly differentiate cytokine secretion among distinct cell types. This approach measures cytokine production rather than release. Additionally, as discussed by de Wolf et al. (2018) (217), effector-released cytokines can be measured at the single-cell level using enzyme-linked immunospot assays (ELISPot). Alternative methods, such as the FluoroSpot assay based on fluorophores, enable the precise detection of multiple cytokines per cell. Furthermore, the LysisPot platform employs target cell lines that express β-galactosidase, an enzyme released upon lysis. This technique enables the characterization of the CAR T-cell product’s direct cytotoxic activity and cytokine (IFN-γ) release at the single-cell level. Another strategy relies on correlating T-cell degranulation with killing activity. Following interaction with target cells, markers of T-cell activation and degranulation (e.g., CD107a) are expressed on the CAR T-cell surface, detectable via flow cytometry (217). Finally, CAR-T cell therapy efficacy *in vivo* can also be predicted by assessing the proliferation capacity of the cell upon target antigen recognition by assessing the incorporation of labelled-DNA thymidine or using fluorescent markers such as CFSE or other CellTrace kit (Thermofisher).

Overall, the phenotypic and functional maturity of the generated iT and CAR iT cell effectors has to be ensured as well as an anti-tumor potential comparable with natural T cells. Moreover, as iPSC/derived CAR T cells are intended to provide an off the shelf therapy product, testing long term stability of the cryopreserved product as well as holding time after thawing is crucial to define it shelf life and ensure efficacy and safety upon infusion (218).

## 10 Conclusions

In conclusion, the clinical production of iCAR T cells represents a remarkable leap forward in the field of cancer immunotherapy. The unique advantages offered by iPSCs, including their potential for greater compliance with good manufacturing practice (GMP) standards, scalability, and ease of genetic manipulation, underscore their importance as a source for CAR T cell generation. Moreover, the concept of universal iPSC clones, which can be standardized and quality-controlled in a manner akin to pharmaceutical production, holds tremendous promise for streamlining the manufacturing process and ensuring product consistency. However, it is clear that the journey towards clinical implementation is not without its challenges.

The complexity of T cell development from iPSCs necessitates ongoing research and novel ideas to streamline and optimize the process. Notable advances have been made in the field of hematopoietic development, in establishing cGMP-compliant and scalable culturing and genetic engineering processes. This part is in many aspects common with the process of NK cell generation from iPSC (iNK), which is more straightforward thus, leading the more rapid clinical application of iNK cell products (NCT05182073, NCT05950334, NCT05336409). In contrast to the generation of NK cells from iPSC, the production of CAR iT cells has a multistep nature, involving various stages from iPSC establishment to hematopoietic and T cell differentiation and multiple potential endpoint T cell phenotypes (CD8, CD4, cytotoxic or regulatory), thus, presenting logistical challenges that demand innovative solutions. To make this therapy more clinically applicable, efforts must focus on manufacturing improvements, such as closed-system processes, enhanced scalability, and process optimization, all of which can contribute to improved affordability and accessibility for patients. A promising direction in this endeavor involves transitioning from modular production systems for each cell process (iPSC, hematopoietic progenitors and T cells) to preferentially adopting all-in-one systems, which can simplify the workflow, reduce production timelines, and enhance the overall efficiency of iPSC-derived CAR T cell manufacturing. This paradigm shift aligns with the overarching goal of expanding the reach of CAR T cell therapy to a broader patient population as an off the shelf therapy.

In summary, while challenges persist, the ongoing advancements in CAR iT cell production and first-in-human clinical application (NCT04629729) offer a tantalizing glimpse into a future where innovative treatments are more accessible, affordable, and effective, paving the way for improved outcomes in cancer immunotherapy.

## Author contributions

RN: Conceptualization, Writing – original draft, Writing – review & editing. LG-P: Conceptualization, Writing – original draft, Writing – review & editing. MT: Conceptualization, Supervision, Writing – review & editing.
